# Synthesis and Electrochemical Properties of LiNi_0.5_Mn_1.5_O_4_ Cathode Materials with Cr^3+^ and F^−^ Composite Doping for Lithium-Ion Batteries

**DOI:** 10.1186/s11671-017-2172-z

**Published:** 2017-06-15

**Authors:** Jun Li, Shaofang Li, Shuaijun Xu, Si Huang, Jianxin Zhu

**Affiliations:** School of Light Industry and Chemical Engineering, Guangdong University of Technology, No.100 Waihuan Xi Road, Guangzhou Higher Education Mega Center, Panyu District, Guangzhou, 510006 Guangdong People’s Republic of China

**Keywords:** Solid-state method, Lithium-ion batteries, LiNi_0.5_Mn_1.5_O_4_, Composite doping

## Abstract

A Cr^3+^ and F^−^ composite-doped LiNi_0.5_Mn_1.5_O_4_ cathode material was synthesized by the solid-state method, and the influence of the doping amount on the material’s physical and electrochemical properties was investigated. The structure and morphology of the cathode material were characterized by XRD, SEM, TEM, and HRTEM, and the results revealed that the sample exhibited clear spinel features. No Cr^3+^ and F^−^ impurity phases were found, and the spinel structure became more stable. The results of the charge/discharge tests, cyclic voltammetry (CV), and electrochemical impedance spectroscopy (EIS) test results suggested that LiCr_0.05_Ni_0.475_Mn_1.475_O_3.95_F_0.05_ in which the Cr^3+^ and F^−^ doping amounts were both 0.05, had the optimal electrochemical properties, with discharge rates of 0.1, 0.5, 2, 5, and 10 C and specific capacities of 134.18, 128.70, 123.62, 119.63, and 97.68 mAh g^−1^ , respectively. After 50 cycles at a rate of 2 C, LiCr_0.05_Ni_0.475_Mn_1.475_O_3.95_F_0.05_ showed extremely good cycling performance, with a discharge specific capacity of 121.02 mAh g^−1^ and a capacity retention rate of 97.9%. EIS test revealed that the doping clearly decreased the charge-transfer resistance.

## Background

The increasing demand for electric vehicles (EV), hybrid electric vehicles (HEV), and high-capacity storage batteries requires higher performance lithium-ion batteries with improved energy density and power density [1–3]. The cathode material is a key material in lithium-ion batteries, and research and development into high-potential cathode materials is one of the main ways to improve the energy density of lithium-ion batteries. Spinel LiNi_0.5_Mn_1.5_O_4_ has the advantage of discharge voltage plateaus at approximately 4.7 V: low cost, excellent structural stability, and heat stability, and is considered one of the most promising cathode materials for lithium-ion batteries. However, the cycling stability of LiNi_0.5_Mn_1.5_O_4_ is poor, and cycling of this material results in the Jahn-Teller effect and Mn dissolution [4–7].

Modification of the material by doping and coating has been applied to suppress the Jahn-Teller effect and to reduce Mn loss in order to improve the electrochemical properties of the material. Doping modification is a very effective approach that can not only enhance the stability of the crystal structure but also improve the rate capability of the material [8, 9]. During charging, 4.7% of the volume of LiNi_0.5_Mn_1.5_O_4_ is maintained when going from the lithium-rich phase to the lithium-poor phase. The volume change in the material during the insertion/extraction process of Li ions can be effectively suppressed by applying a small amount of doping and surface coating, and furthermore, doping can improve the rate capability and cycling performance of the material [10–12]. Cation doping ( Na [13], Ru [14], Rh [15], Co [16], Al [17], Cr [18], Zn [19], Nd [20], Mg [21], Mo [22], Sm [23], Cu [24], etc.) and anion doping (S [25], P [26], and F [27]) have been applied to modify LiNi_0.5_Mn_1.5_O_4._ For instance, compared to pure LiNi_0.5_Mn_1.5_O_4_, Al-doped LiNi_0.5_Mn_1.5_O_4_ can effectively improve the discharge capacity (up to 140 mAh g^−1^) and cycling stability (70% capacity retention after 200 cycles) [28].

In this paper, F^-^ and Cr^3+^ are selected to improve the rate capability via anion-cation compound substitution, and their doping amounts are optimized [29]. In addition, the structure, morphology, and electrochemical properties of the samples were tested and analyzed.

## Methods

The LiNi_0.5_Mn_1.5_O_4_ materials were synthesized by the solid-state method using Ni(CH_3_COO)_2_ · 4H_2_O、Mn(CH_3_COO)_2_ · 4H_2_O and Cr(CH_3_COO)_3_ · 6H_2_O as the starting materials.

## Experimental

### Preparation of LiCr_x_Ni_0.5−0.5x_Mn_1.5−0.5x_O_3.95_F_0.05_

The LiNi_0.5_Mn_1.5_O_4_ materials were synthesized by the solid-state method using Ni(CH_3_COO)_2_ · 4H_2_O、Mn(CH_3_COO)_2_ · 4H_2_O and Cr(CH_3_COO)_3_ · 6H_2_O as the starting materials. The materials were fully mixed by ball-milling for 2 h using stoichiometric amounts of LiCr_x_Ni_0.5−0.5x_Mn_1.5−0.5x_O_3.95_F_0.05_ (*x* = 0.025, 0.05, 0.075), and the dry mixture was heated at 400 °C in air for 5 h. The Ni-Mn-Cr complex oxide formed after natural cooling in a muffle furnace. The obtained complex oxide and lithium source (Li_2_CO_3_ and LiF) were mixed by ball-milling for 4 h using anhydrous alcohol as a dispersant, and the mixture was then heated at 850 °C in air for 12 h to strengthen its crystallization in a muffle furnace. After being reduced at 650 °C in air for 12 h, materials with different Cr^3+^ and F^−^ composite doping amounts, LiCr_x_Ni_0.5−0.5x_Mn_1.5−0.5x_O_3.95_F_0.05_ (*x* = 0.025, 0.05, 0.075), were obtained after natural cooling in a muffle furnace.

### Characterization

The crystal structures of the samples were identified by X-ray diffraction (XRD, UltimaIII, diffractometer Cu-Kα radiation, 40 kV, 40 mA, Rigaku, Japan) at room temperature over a 2θ range of 10° to 80° with a scanning speed of 8° min^−1^. The morphology of the LiCr_x_Ni_0.5−0.5x_Mn_1.5−0.5x_O_3.95_F_0.05_ samples was measured by a scanning electron microscopy (SEM, Hitachi, S-3400N, Japan). The microstructure and elemental composition of the obtained materials were observed by transmission electron microscopy (TEM, Tecnai G2 F20, FEI) equipped with energy dispersive spectroscopy (EDS).

### Electrochemical Performance Test

The electrochemical properties were assessed with CR2032 coin cells, and the cells consisted of the LiCr_x_Ni_0.5−0.5x_Mn_1.5−0.5x_O_3.95_F_0.05_ electrode as the cathode electrode, Li metal foil as the anode electrode, American Celgard2400 as the separator and 1 mol/L LiPF_6_ in EC/EMC/DMC (1:1:1 in volume) as the electrolyte. The cathode was synthesized by mixing the active material, carbon black, and polyvinylidene fluoride (PVDF) at a weight ratio of 8:1:1 in the N-methyl pyrrolidinone (NMP) to form a homogeneous slurry, which was then coated on Al foil by a doctor blade coater and subsequently dried in a vacuum oven at 120 °C for 24 h to remove NMP and residual water. The coin cells were assembled in an argon-filled glove box (MBRAUN PRS405/W11006-1, Germany).

The electrochemical performance of LiCr_x_Ni_0.5−0.5x_Mn_1.5−0.5x_O_3.95_F_0.05_/Li coin cells was evaluated by charging and discharging over 3.5–5.0 V using a CT-300-1A-SA tester (Neware Technology Ltd.). Cyclic voltammograms (CV) tests (the cathode was the working electrode and Li metal foil was both the counter and reference electrode) were carried out using an electrochemical work station (Metrohm Co., Autolab PGSTAT302N, Netherlands) with a scanning rate of 0.1 mV/s and a scanning frequency of 0.5 Hz between 3.5 and 5.0 V. Electrochemical impedance spectroscopy (EIS) was conducted on an electrochemical work station with an AC amplitude of 5 mV in the scanning frequency range of 0.01 to 100 kHz (the cathode was the working electrode and Li metal foil was both the counter and reference electrode ).

## Results and Discussion

**Fig. 1:**
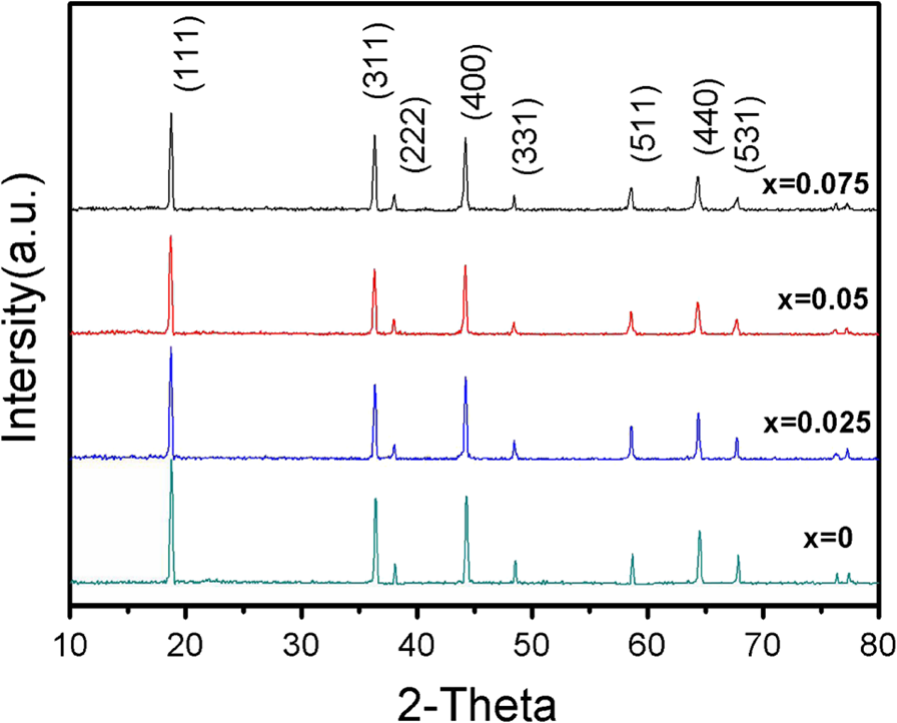
XRD patterns of LiNi_0.5_Mn_1.5_O_4_ and LiCr_x_Ni_0.5−0.5x_Mn_1.5−0.5x_O_3.95_F_0.05_ (*x* = 0.025, 0.05, 0.075)

Figure 1 shows the XRD pattern of the LiNi_0.5_Mn_1.5_O_4_ and LiCr_x_Ni_0.5−0.5x_Mn_1.5−0.5x_O_3.95_F_0.05_ (*x* = 0.025, 0.05, 0.075) materials. The pattern revealed that the Cr^3+^ and F^−^ compound-doped materials had the same diffraction peaks as the undoped sample, suggesting that the samples were synthesized without impurity phases and that Cr^3+^ and F^−^ compound doping would not change the spinel crystal structure. No impurity peaks or superstructure peaks were found, indicating that some of the Ni^2+^, Mn^4+^, Mn^3+^, and O^2−^ atoms in the spinel phase were successfully substituted by Cr^3+^ and F^−^. The strength of the diffraction peaks of the Cr^3+^-doped LiCr_x_Ni_0.5−0.5x_Mn_1.5−0.5x_O_3.95_F_0.05_ samples decreased, and excess dopants concentration influenced the degree of crystallinity. The lattice parameters for the LiNi_0.5_Mn_1.5_O_4_ and LiCr_x_Ni_0.5−0.5x_Mn_1.5−0.5x_O_3.95_F_0.05_ (*x* = 0.025, 0.05, 0.075) materials were calculated by Jade5.0, and the results are shown in Table 1.

**Table 1:** Refinement results for the samples

Samples	a/Å
LiNi_0.5_Mn_1.5_O_4_	8.1775
LiCr_0.025_Ni_0.4875_Mn_1.4875_O_3.95_F_0.05_	8.1803
LiCr_0.05_Ni_0.475_Mn_1.475_O_3.95_F_0.05_	8.1839
LiCr_0.075_Ni_0.4625_Mn_1.4625_O_3.95_F_0.05_	8.1821

**Fig. 2:**
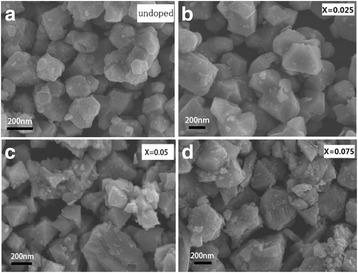
SEM images of LiCr_x_Ni_0.5−0.5x_Mn_1.5−0.5x_O_3.95_F_0.05_.(**a**) *x* = 0, (**b**) *x* = 0.025, (**c**) *x* = 0.05, (**d**) *x* = 0.075

Figure 2 shows the SEM images of the LiNi_0.5_Mn_1.5_O_4_ and LiCr_x_Ni_0.5−0.5x_Mn_1.5−0.5x_O_3.95_F_0.05_ (*x* = 0.025, 0.05, 0.075) with ×10000 magnification. The LiNi_0.5_Mn_1.5_O_4_ sample consists of uniform, submicron-sized particles, and the crystals have a quasi-octahedral shape. After Cr^3+^ and F^−^ compound doping, the LiCr_x_Ni_0.5−0.5x_Mn_1.5−0.5x_O_3.95_F_0.05_ (*x* = 0.025, 0.05, 0.075) samples exhibited highly crystalline particles and a typical spinels with an octahedral shape and sharp edges and corners.

**Fig. 3:**
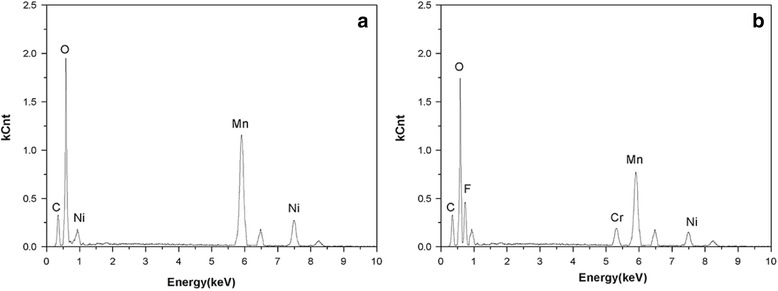
EDS patterns of LiNi_0.5_Mn_1.5_O_4_ (**a**) and LiCr_0.05_Ni_0.475_Mn_1.475_O_3.95_F_0.05_ (**b**)

**Fig. 4:**
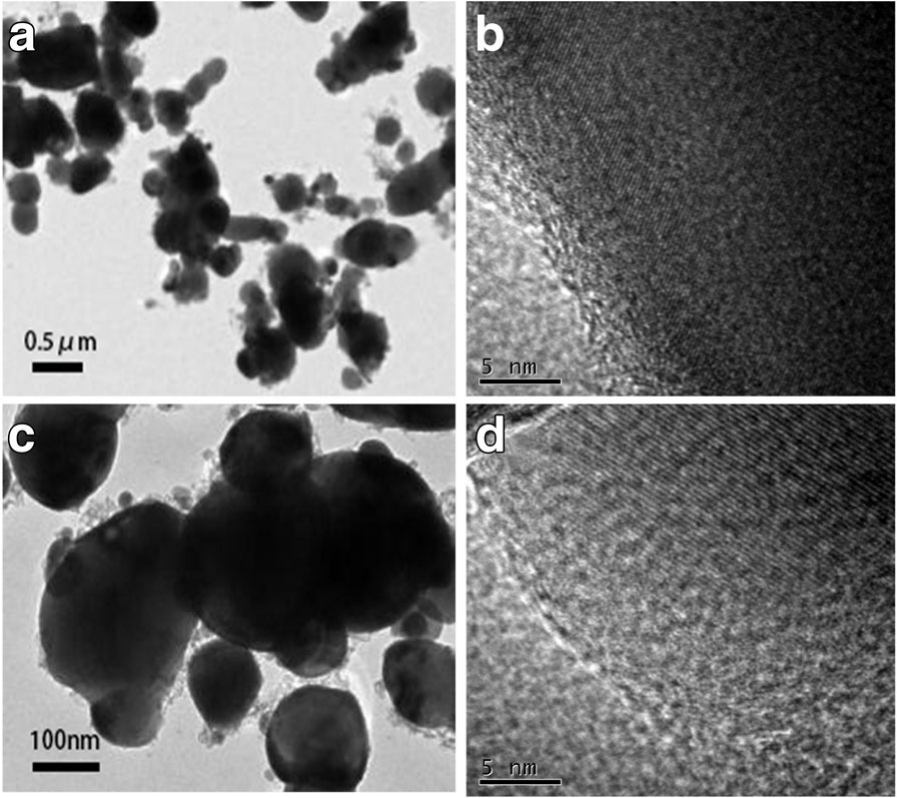
TEM and HRTEM images of LiNi_0.5_Mn_1.5_O_4_ (**a** and **b**) and LiCr_0.05_Ni_0.475_Mn_1.475_O_3.95_F_0.05_ (**c** and **d**)

The existence of chromium and fluoride in the spinel LiCr_0.05_Ni_0.475_Mn_1.475_O_3.95_F_0.05_ compound was verified by EDS, as shown in Fig. 3. The TEM and high-resolution TEM (HRTEM) images of the crystal morphology and lattice fringes are shown in Fig. 4. Both LiNi_0.5_Mn_1.5_O_4_ and LiCr_0.05_Ni_0.475_Mn_1.475_O_3.95_F_0.05_ showed similar surface morphologies. The distance between the lattice fringes for LiNi_0.5_Mn_1.5_O_4_ was measured to be 0.4835 nm, corresponding to the (111) plane of spinel. After doping, the lattice spacing in Fig. 4d reveals a value of 0.4811 nm, indicating that the higher bonding energy of Cr-O may shrink the spinel framework. Therefore, LiCr_0.05_Ni_0.475_Mn_1.475_O_3.95_F_0.05_ is expected to have excellent electrochemical properties for lithium storage.

**Fig. 5:**
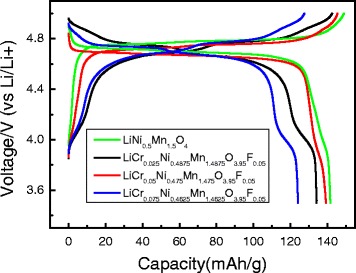
Charge/discharge curves of LiNi_0.5_Mn_1.5_O_4_ and LiCr_x_Ni_0.5−0.5x_Mn_1.5−0.5x_O_3.95_F_0.05_ (*x* = 0.025, 0.05, 0.075) at 0.1 C

Figure 5 displays the charge/discharge curves of the LiNi_0.5_Mn_1.5_O_4_ and LiCr_x_Ni_0.5−0.5x_Mn_1.5−0.5x_O_3.95_F_0.05_ (*x* = 0.025, 0.05, 0.075) samples, where the cells were tested over a potential range of 3.5–5.0 V at a rate of 0.1 C. The discharge specific capacities of the LiNi_0.5_Mn_1.5_O_4_ and LiCr_x_Ni_0.5−0.5x_Mn_1.5−0.5x_O_3.95_F_0.05_ (*x* = 0.025, 0.05, 0.075) samples were 141.59, 139.38, 134.18, and 124.47 mAh g^−1^ at 0.1 C, respectively. The charge/discharge curve of the doped samples was composed of two obvious voltage plateaus at approximately 4.7 and 4.1 V. The voltage plateau at approximately 4.7 V was attributed to the Ni^2+^/Ni^4+^ redox couple, while the small voltage plateau at approximately 4.1 V may be due to the substitution of F^−^ for O^2−^, which reduced the amount of negative charge and changed the valence of the transition metal (Mn^4+^ was reduced to Mn^3+^) in order to maintain charge balance.

**Fig. 6:**
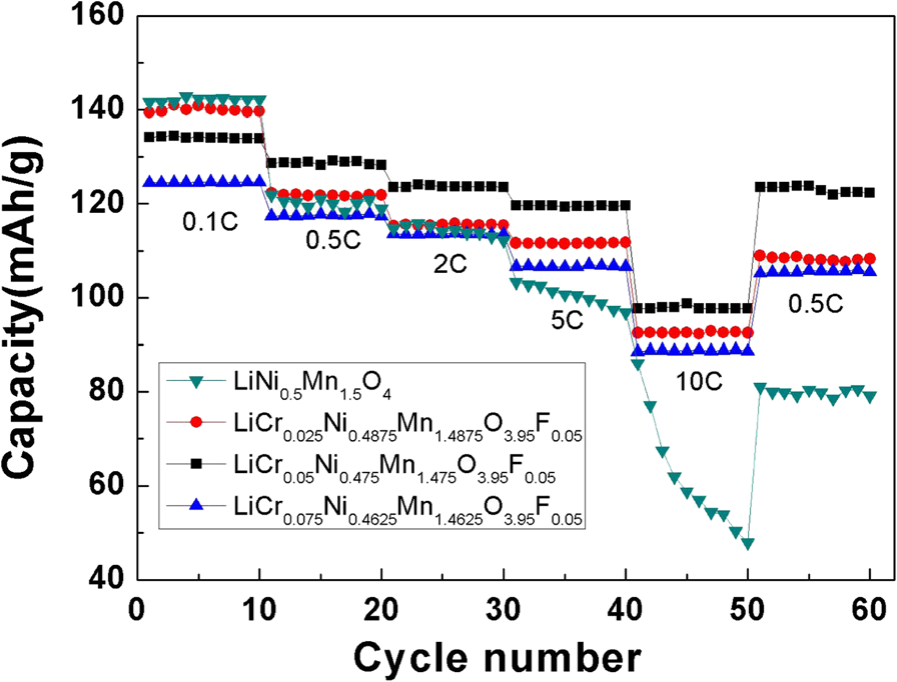
Cycling performance of the LiNi_0.5_Mn_1.5_O_4_ and LiCr_x_Ni_0.5−0.5x_Mn_1.5−0.5x_O_3.95_F_0.05_ (*x* = 0.025, 0.05, 0.075) at different rates

The rate capability is very important for lithium-ion batteries. The cycling performance curves of the LiNi_0.5_Mn_1.5_O_4_ and LiCr_x_Ni_0.5−0.5x_Mn_1.5−0.5x_O_3.95_F_0.05_ (*x* = 0.025, 0.05, 0.075) samples at different rates are shown in Fig. 6. The highest specific discharge capacity at 0.1 C was observed for LiNi_0.5_Mn_1.5_O_4_ (141.59 mAh g^−1^), and the second highest discharge capacity was observed for LiCr_0.025_Ni_0.4875_Mn_1.4875_O_3.95_F_0.05_ (139.38 mAh g^−1^). However, at other high rates of 0.5, 2, 5, and 10 C, the specific discharge capacities of the LiCr_0.05_Ni_0.475_Mn_1.475_O_3.95_F_0.05_ were the highest, which were 128.70, 123.62, 119.63, and 97.68 mAh g^−1^, respectively. When undoped LiNi_0.5_Mn_1.5_O_4_ is discharged at a rate of 2 C , the attenuation of its specific discharge capacity is more obvious. At a discharge rate of 5 C , the structure of the materials may be severely damaged. As the doping amount and substitution of Cr^3+^ increases, the cycling stability increases. A higher doping amount will reduce the specific discharge capacity of the material, making the 4.1 V plateau more obvious and decreasing the energy density of the batteries. On one hand, due to the small polarization at low rate, the polarization effect showed little differences before and after doping. However, the amount of active material decreased after doping, resulting in lower specific capacity. On the other hand, owing to the large polarization at high rate and improved lithium-ion diffusion coefficient, the doped LiNi_0.5_Mn_1.5_O_4_ cathode exhibited higher specific capacity. This result indicates that an appropriate amount of Cr^3+^, F^−^ co-doping can lead to excellent cycling stability and rate capacity.

**Fig. 7:**
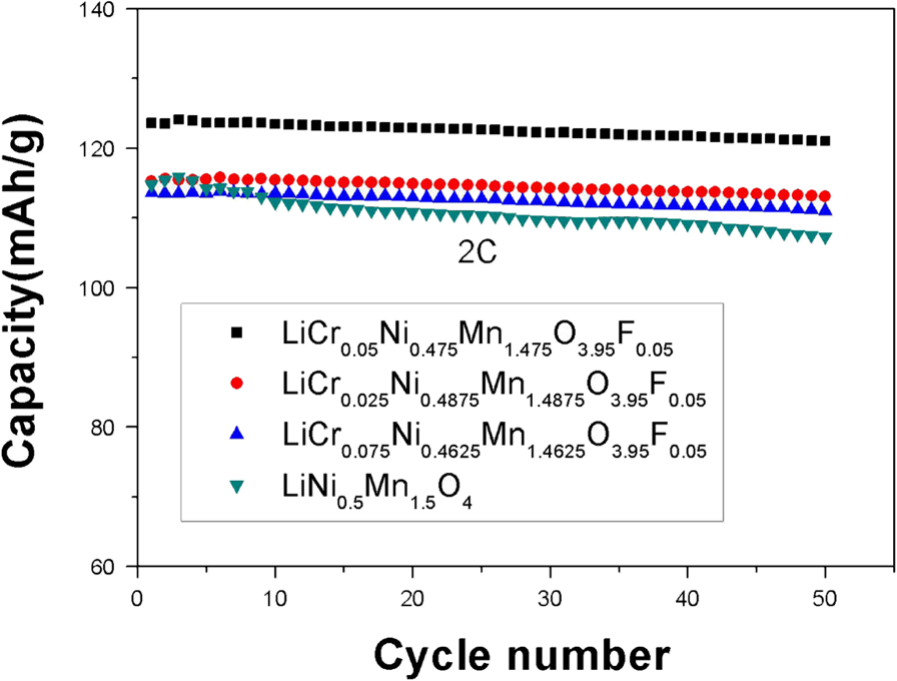
Cycling performance of LiNi_0.5_Mn_1.5_O_4_ and LiCr_x_Ni_0.5−0.5x_Mn_1.5−0.5x_O_3.95_F_0.05_ (*x* = 0.025, 0.05, 0.075) at 2 C

Figure 7 clearly shows the cycling performance of the LiNi_0.5_Mn_1.5_O_4_ and LiCr_0.05_Ni_0.475_Mn_1.475_O_3.95_F_0.05_ samples after 50 cycles at 2 C. The initial discharge capacities of LiCr_0.05_Ni_0.475_Mn_1.475_O_3.95_F_0.05_ and LiNi_0.5_Mn_1.5_O_4_ were 123.62 and 114.77 mAh g^−1^, respectively, indicating that LiCr_0.05_Ni_0.475_Mn_1.475_O_3.95_F_0.05_ has a higher initial discharge capacity than undoped LiNi_0.5_Mn_1.5_O_4_. Consequently, LiCr_0.05_Ni_0.475_Mn_1.475_O_3.95_F_0.05_ could deliver a reversible discharge capacity of 121.02 mAh g^−1^ with a capacity retention of 97.9% after 50 cycles, while LiNi_0.5_Mn_1.5_O_4_ only maintained a reversible discharge capacity of 106.24 mAh g^−1^ with a capacity retention of 92.6%. The capacity retentions of LiCr_0.025_Ni_0.4875_Mn_1.4875_O_3.95_F_0.05_ and LiCr_0.075_Ni_0.4625_Mn_1.4625_O_3.95_F_0.05_ were 95.0 and 94.5%, respectively, which indicates that LiCr_0.05_Ni_0.475_Mn_1.475_O_3.95_F_0.05_ has good capacity retention at high rates among all the samples. The doped material has a higher capacity retention rate due to the bonding energy of Cr-O, which is stronger than the bonding energy of Ni-O and Mn-O and stabilizes the spinel structure. Moreover, the seizing electronic capacity of F^−^ was stronger and more stable after bonding with Ni, Mn, and Cr, thus improving the stability of the spinel structure. Meanwhile, doping also reduced the erosion of the material by HF in the electrolyte solution and the irreversible loss of active substance during the cycling process. Wang et al. [30] reported that LiNi_0.4_Cr_0.15_Mn_1.45_O_4_ can deliver a reversible discharge capacity of 139.7 mAh g^−1^ after 40 cycles, corresponding a capacity retention of 97.08%. Li et al. [31] reported the initial discharge capacities of LiNi_0.5_Mn_1.5_O_3.9_F_0.1_ at 0.1, 0.5, 1, 2, and 5 C were 129.07, 123.59, 118.49, 114.49, and 92.57 mAh g^−1^, respectively.

**Fig. 8:**
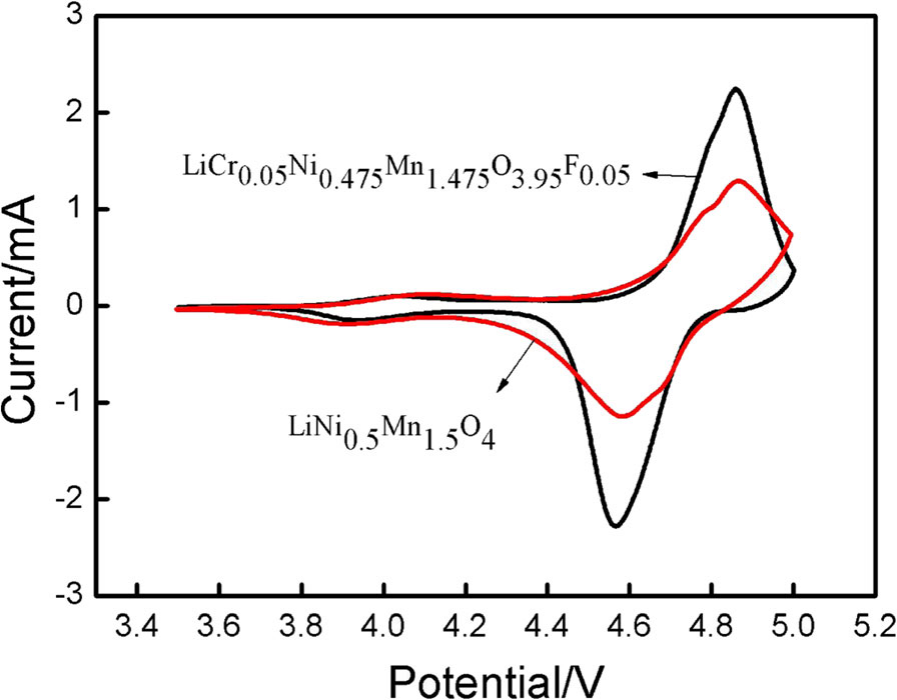
Cyclic voltammogram of LiNi_0.5_Mn_1.5_O_4_ and LiCr_0.05_Ni_0.475_Mn_1.475_O_3.95_F_0.05_

A more detailed analysis of the electrochemical performance was performed by CV and EIS. Figure 8 shows the CV curves of LiCr_0.05_Ni_0.475_Mn_1.475_O_3.95_F_0.05_ and pure phase LiNi_0.5_Mn_1.5_O_4_. The potential difference of these two materials was 0.298 V. The LiNi_0.5_Mn_1.5_O_4_ oxidation peak potential was 4.861 V, while the reduction peak potential was 4.563 V. The oxidation peak current (*I*_Pa_) was 2.242 mA, and the reduction peak current (*I*_Pc_) was 2.288 mA, and thus the *I*_Pa_/*I*_Pc_ ratio was 0.9799. The LiCr_0.05_Ni_0.475_Mn_1.475_O_3.95_F_0.05_ oxidation peak potential was 4.864 V, the reduction peak potential was 4.578 V, and the potential difference was 0.286 V. The *I*_Pa_ was 1.273 mA, the *I*_Pc_ was 1.277 mA, and the *I*_Pa_/*I*_Pc_ ratio was 0.9968 (approximately 1). The above results indicated that the co-doped materials had good reversibility of lithium ions intercalation/deintercalation and improved coulombic efficiency.

**Fig. 9:**
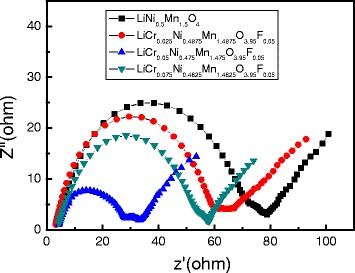
EIS patterns of LiNi_0.5_Mn_1.5_O_4_ and LiCr_x_Ni_0.5−0.5x_Mn_1.5−0.5x_O_3.95_F_0.05_ (*x* = 0.025, 0.05, 0.075)

Figure 9 shows the EIS patterns of the samples. All the EIS spectra in the figure consist of two semicircles in the high-to-medium frequency region and a sloping line in the low frequency region. The semicircle in the high-frequency region corresponds to lithium ions passing through the electrolyte surface film resistance (*R*_s_). The semicircle in the medium frequency region corresponds to the charge-transfer resistance (*R*_ct_), and the sloping line in the low frequency region is the Warburg impedance (*Z*_w_), which is related to lithium ion diffusion in the materials. As seen in Fig. 7, doping decreased the *R*_s_, which contributed to improving the diffusivity of lithium ions, the conductivity, and the rate capability of the materials.

**Fig. 10:**
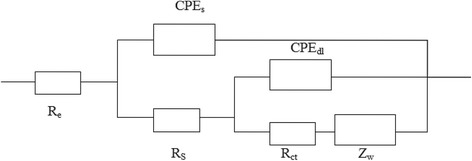
The equivalent circuit for the Nyquist plots

The Nyquist plot of the equivalent circuit analog fitted by the ZsimpWin software is shown in Fig. 10. In this circuit, *R*_e_ and *R*_s_ represent the electrolyte resistance and the particle-to-particle interfacial contact resistance of the SEI film. *R*_ct_ is the charge-transfer resistance, and *Z*_w_ stands for the Warburg impedance caused by diffusion of lithium ions. CPEs and CPEdl are constant phase elements of the solid electrolyte membrane and the double-layer capacitance of the electrode-electrolyte interface, respectively [32]. The fitting parameters of the equivalent circuit analog are summarized in Table 2.

**Table 2:** Fitting parameters of the Nyquist plots for the samples

Samples	Rs/Ω	Rct/Ω	D/cm^2^ s^-1^
LiNi_0.5_Mn_1.5_O_4_	5.2	70.3	1.05 × 10^−11^
LiCr_0.025_Ni_0.4875_Mn_1.4875_O_3.95_F_0.05_	7.9	57.6	5.48 × 10^−11^
LiCr_0.05_Ni_0.475_Mn_1.475_O_3.95_F_0.05_	2.9	24.9	1.51 × 10^−10^
LiCr_0.075_Ni_0.4625_Mn_1.4625_O_3.95_F_0.05_	4.3	50.2	4.49 × 10^−11^

Previous studies have suggested that the diffusion coefficient of lithium ions is associated with the Warburg factor, which can be calculated by the sloping line in the low frequency region. The lithium-ions diffusion coefficient was calculated by Fick’s rule using the following equation: [33]

1 $$ D=\frac{R^2{T}^2}{2{A}^2{n}^4{F}^4{C}^2{\sigma}^2} $$

where *D* is the lithium-ion diffusion coefficient, *T* is the absolute temperature, *R* is the gas constant, *A* is the surface area of the electrode, *n* is the electron transfer number, *F* is the Faraday constant, *C* is the molar concentration of lithium ions, and σ is the Warburg factor, which is the slope of the sloping line in Fig. 7.

As seen in Table 2, the *R*_s_ values of the doped samples were greatly decreased compared with the undoped sample, and the *R*_s_ value of LiCr_0.05_Ni_0.475_Mn_1.475_O_3.95_F_0.05_ decreased greatly. The decrease in the *R*_s_ value indicates that Cr^3+^, F^−^ co-doping can inhibit the growth of the SEI film to some extent, which may be due to the F^−^ side reactions between the electrode material and the electrolyte solution. A lower charge-transfer resistance value indicates lower electrochemical polarization, which will lead to higher rate capability and cycling stability. LiCr_0.05_Ni_0.475_Mn_1.475_O_3.95_F_0.05_ exhibited the lowest *R*_ct_ value (24.9 Ω) and the highest lithium diffusion coefficient (1.51 × 10^−10^ cm^2^ s^−1^) among all the samples, indicating that its electrochemical polarization is the lowest and the lithium-ion mobility of LiNi_0.5_Mn_1.5_O_4_ can be effectively improved by anion-cation compound substitution. EIS also can be used to compare the size of the electronic conductivity. The smaller charge-transfer resistance of the Cr^3+^ and F^−^ co-doping LiNi_0.5_Mn_1.5_O_4_ indicates a larger electronic conductivity than that of pristine LiNi_0.5_Mn_1.5_O_4_. The electronic conductivity of LiNi_0.5_Mn_1.5_O_4_ is about 3.88 × 10^−5^ S cm^−1^, while the electronic conductivities of LiCr_x_Ni_0.5−0.5x_Mn_1.5−0.5x_O_3.95_F_0.05_ (*x* = 0.025, 0.05, 0.075) samples were 6.19 × 10_−_^5^ S cm^-1^, 1.25 × 10^-4^ S cm^−1^, and 5.98 × 10^−5^ S cm^−1^, respectively. In fact, LiCr_0.05_Ni_0.475_Mn_1.475_O_3.95_F_0.05_ has the best electrochemical performance among all four samples. The decrease in *R*_ct_ and the increase in *D* indicate that the proper amount of Cr^3+^, F^−^ co-doping has a positive effect on the electrochemical reaction activity of the material.

## Conclusions

The Cr^3+^, F^−^ co-doped analog of LiNi_0.5_Mn_1.5_O_4_ (LiCr_x_Ni_0.5−0.5x_Mn_1.5−0.5x_O_3.95_F_0.05_ (*x* = 0.025, 0.05, 0.075)) was synthesized by the high-temperature solid-state method. The materials’s XRD patterns showed that Cr^3+^ and F^−^ successfully substituted some of the Ni^2+^, Mn^4+^, Mn^3+^, and O^2-^ atoms in the spinel material, and no impurity peaks existed. The specific discharge capacities of LiCr_0.05_Ni_0.475_Mn_1.475_O_3.95_F_0.05_ at 0.1, 0.5, 2, 5, and 10 C were 134.18, 128.70, 123.62, 119.63, and 97.68 mAh g^−1^, respectively. The specific discharge capacity was 121.02 mAh g^−1^ after 50 cycles at 2 C, which is of 97.9% the initial discharge capacity. The capacity retention rate of LiCr_0.05_Ni_0.475_Mn_1.475_O_3.95_F_0.05_ was the largest among the samples. The materials had good crystallinity, and the largest number of octahedral spinel was well distributed. Cr^3+^, F^−^ co-doped of the materials significantly improved the specific discharge capacity at higher rate, improved the cycling stability, enhanced the reversibility of lithium ions, and reduced the impedance value.
